# Ceramide Synthases Expression and Role of Ceramide Synthase-2 in the Lung: Insight from Human Lung Cells and Mouse Models

**DOI:** 10.1371/journal.pone.0062968

**Published:** 2013-05-14

**Authors:** Irina Petrache, Krzysztof Kamocki, Christophe Poirier, Yael Pewzner-Jung, Elad L. Laviad, Kelly S. Schweitzer, Mary Van Demark, Matthew J. Justice, Walter C. Hubbard, Anthony H. Futerman

**Affiliations:** 1 Division of Pulmonary and Critical Care Medicine, Department of Medicine, Indianapolis, Indiana, United States of America; 2 Richard L. Roudebush Veteran Affairs Medical Center, Indianapolis, Indiana, United States of America; 3 Department of Biological Chemistry, Weizmann Institute of Science, Rehovot, Israel; 4 Department of Clinical Pharmacology, Johns Hopkins University, Baltimore, Maryland, United States of America; National Jewish Health, United States of America

## Abstract

Increases in ceramide levels have been implicated in the pathogenesis of both acute or chronic lung injury models. However, the role of individual ceramide species, or of the enzymes that are responsible for their synthesis, in lung health and disease has not been clarified. We now show that C24- and C16-ceramides are the most abundant lung ceramide species, paralleled by high expression of their synthetic enzymes, ceramide synthase 2 (CerS2) and CerS5, respectively. Furthermore, the ceramide species synthesis in the lung is homeostatically regulated, since mice lacking very long acyl chain C24-ceramides due to genetic deficiency of CerS2 displayed a ten-fold increase in C16-ceramides and C16-dihydroceramides along with elevation of acid sphingomyelinase and CerS5 activities. Despite relatively preserved total lung ceramide levels, inhibition of de novo sphingolipid synthesis at the level of CerS2 was associated with significant airflow obstruction, airway inflammation, and increased lung volumes. Our results suggest that ceramide species homeostasis is crucial for lung health and that CerS2 dysfunction may predispose to inflammatory airway and airspace diseases.

## Introduction

**Figure 1:**
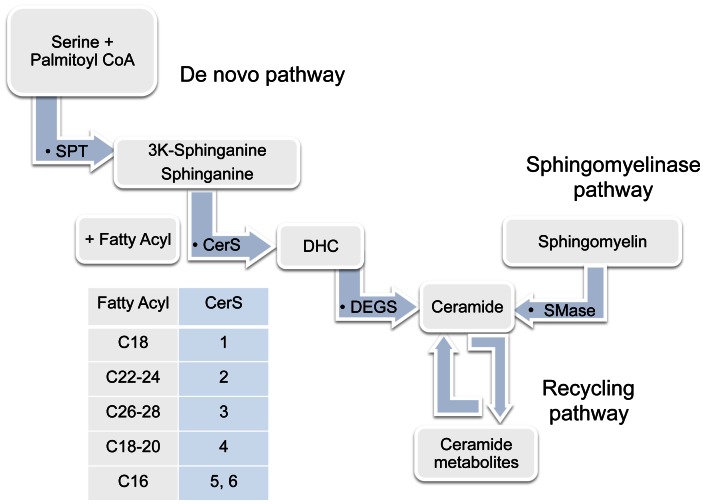
Ceramide metabolic pathways. Ceramide can be synthesized via the de novo pathway regulated by serine palmitoyl transferase (SPT), ceramide synthases (CerS; isoforms and their preferred substrates described in tabular format), and desaturases (DEGS); via sphingomyelinase pathway regulated by sphingomyelinases (SMases); or via the recycling pathway.

Ceramide, a signaling sphingolipid involved in cell differentiation and apoptosis, has received great attention recently due to reports of abnormal ceramide accumulation in prevalent lung diseases such as acute lung injury, cystic fibrosis, or chronic obstructive pulmonary disease (COPD). Furthermore, the de novo pathway of ceramide synthesis has been implicated in asthma. Ceramide, which consists of multiple molecular species distinguished by fatty acyl chain length, saturation, and α-hydroxylation, is synthesized by a family of ceramide synthases (CerS). Six CerS exist, each using defined acyl chains for synthesis of dihydroceramides (DHCer) and ceramides. Thus, CerS1 uses mostly C18-CoA, CerS2 uses C22 to C24-CoAs, CerS3 uses C26 and higher acyl CoAs [1], CerS4 uses C18- and C20-CoAs, and CerS5 and CerS6 use mostly C16-CoA [2] (Fig. 1). These CerS have defined tissue distribution [3]. For instance, lung epithelial cells exhibit high levels of CerS5 expression, but little is known about CerS' role in the lung, in general. To date, the role of specific ceramides in lung function has not been addressed. The goal of our study was to investigate the CerS expression profile and the role of CerS2 in the lung.

The pathways by which ceramides are synthesized intracellular include sphingomyelin hydrolysis performed by acid or neutral sphingomyelinases, and de novo synthesis, which requires serine palmitoyl transferase (SPT) activation, itself regulated by ORMDL proteins [4], followed by CerS activation to generate dihydroceramide, which is then desaturated to ceramide. The metabolism of ceramide either by deacylation to sphingosine or by glycosylation to glycosylated ceramides can itself be harnessed in a recycling fashion to re-synthesize or deglycosylate to ceramides, respectively [5] (Fig. 1). Although there might be acyl-chain type preference in the action of several of ceramide generating enzymes, CerS are primarily responsible for ceramide species-specificity. Understanding the role of specific CerS in lung biology is important, given the increasing appreciation of ceramide species-specific cellular function [6]–[8] and the potential need for selective targeting of only deleterious ceramide species. Recently, several groups, including ours, used molecular approaches to individually inhibit the expression of CerS, in order to understand their function in vivo, in various organs. We created a CerS2-null mouse which is unable to synthesize very long acyl chain (VLC) ceramides. These mice are characterized by liver pathology and deficient myelin maintenance in the brain [9]. The impact of loss of any CerS, including CerS2 on the murine lung pathology or function has not yet been described. We hypothesized that because of the central role of ceramides in sphingolipid metabolism, and the importance of ceramide and its various metabolites in cell maintenance and immune regulation, mice deficient in CerS2 will exhibit abnormal lung pathophysiology. Understanding the impact of CerS2 on lung biology will be useful in understating the regulation of sphinoglipid metabolism in the lung in general, and in the future design of therapies that target various ceramide species and metabolites.

In the current study, we determined the expression of ceramide species and CerS in the lung and in principal alveolar cells, and examined the importance of CerS2 in lung function. We demonstrate that CerS2 is essential for proper lung sphingolipid homeostasis and airway function.

## Materials and Methods

### Chemicals and Reagents

All chemicals and reagents were from Sigma-Aldrich (St. Louis, MO), unless otherwise stated.

### Cell Culture

Beas2B cells, a transformed human bronchial cell line, were a kind gift from Dr. Augustine Choi, Harvard University and were originally purchased from American Type Culture Collection (ATCC, Manassas, VA). They were used from passages 5–12. Primary human small airway epithelial cells (SAEC) and human lung microvascular endothelial cells were from Lonza, Walkersville, MD and were maintained up to passage 5 in complete culture medium consisting of EGM-2MV, supplemented with specific SingleQuots® (Lonza, Walkersville, MD).

### Animals

Animal studies were approved by the Institutional Animal Care and Use Committee at Indiana University (Indianapolis, IN) and at the Weizmann Institute of Science (Rehovot, Israel). CerS2 null mice were generated in our laboratory, as previously described [10], using CerS2 genetrap ES cells (Cers2^Gt(S16-4B1)Sor^) obtained from Bay Genomics. CerS2^GT/+^ (denoted in our manuscript CerS^−/+^) mice were intercrossed to generate CerS2^GT/GT^ (denoted in our manuscript as CerS2-null) mice. CerS2-null mice were born in normal mendelian distribution. Experiments were performed in mice (both females and males) age 3–6 months, unless otherwise noted. To inhibit CerS pharmacologically, mice were administered fumonisin B1 (FB1) intraperitoneal (i.p.) (1.1 or 2.2 mg/kg [11]) daily, for 3 days.

### Lung Function Measurement

Mice were anesthetized with ketamine followed by 5% isoflurane inhalation (5%), and were intubated via a custom-made laryngoscope blade. Animals were mechanically ventilated with a rodent ventilator using room air, at a rate of 140 breaths per min, a tidal volume of 0.3 ml, and 5 cm H_2_O of positive end-expiratory pressure. Mice were placed on a heated (37°C) pad and pulmonary function tests were performed with the FlexiVent system (Scireq, Montreal, PQ, Canada).

### Bronchoalveolar Lavage

Bronchoalveolar lavage (BAL) fluid was collected by lavaging the lungs with three aliquots of 1 ml of saline. Samples were centrifuged (6 min, 500×g, 4°C). Cell pellets were collected in 1 ml of red blood cell lysis buffer and following washing, were resuspended in PBS and counted in a hemocytometer. Cytospin slides containing 10,000 cells were prepared using a 3-step stain set (Richard Allen Scientific). Slides were scored by an investigator blinded to the identity of the experimental groups.

### Lung Tissue Harvesting

Following euthanasia, lungs were flushed by perfusing 2×10 ml of saline through the pulmonary circulation. The right bronchus was ligated and a pre-warmed solution of low melting point agarose (0.25% (v/v) in 10% (v/v) formalin/PBS) was slowly introduced into the left lung under a constant pressure of 20 cm H_2_O. The lungs and the heart were dissected en block and cooled on ice for 5–10 min. The right lung was dissected and snap-frozen in liquid N_2_. The left lung was sectioned in the coronal plane into 5 pieces, transferred into a plastic fixation cassette and stored in 10% (v/v) formalin, followed by paraffin embedding. For some experiments, the left lung was inflated with and embedded in optimal cutting temperature compound, frozen in liquid N_2_ and stored at −80°C for preparation of frozen sections.

### SL Analyses

Ceramides, in particular C14:0, C16:0, C18:0, C18:1, C20:0, C24:0, and C24:1 ceramides and dihydroceramides were identified and quantified by liquid chromatography/tandem mass spectrometry on lipid extracts from cells or tissue homogenates and normalized to lipid phosphorus, as described in detail in [12]. ASM and CerS 5/6 activities were measured as previously reported [11], [13]. Briefly, we used the Amplex Red Sphingomyelinase Assay Kit (Molecular Probes, Eugene, OR), following manufacturers protocol. Tissues were homogenized in lysis buffers composed of: 0.2% TritonX-100; 100 mM sodium acetate (pH 5.0); 2 mM EDTA; 0.1 mM Na_3_VO_4_; 1 mM PMSF; 10 µl/ml aprotinin; 10 µl/ml leupeptin. The ASM kinetics was measured using a fluorescence microplate reader. Te lysis buffer used for CerS5/6 activity assays consisted of 5 mM EGTA; 25 mM Hepes pH 7.4; 50 mM NaF; 1 µg/ml Leupeptin; and 10 µg/ml Soybean trypsin inhibitor. The assay was conducted using D-erythro-sphinganine (C16 dihydrosphingosine, Avanti) was resuspended in assay buffer, containing 2 mM MgCl_2_; 20 mM Hepes; 0.5 mM DTT; and 20 mM defatted BSA, and “cold’ palmitoyl-CoA, and ^14^C palmitoyl-CoA (American Radiolabeled Chemicals). After 1 h incubation at 37°C samples were dried under N_2,_ resuspended in in 20 µl chloroform and methanol (1∶1) containing 1 mg/ml bovine brain ceramide and 1 mg/ml diacylglycerol, and loaded onto silica TLC plates. Liquid chromatography was performed in TLC solvent, containing chloroform, methanol and 3.5 N aqueous ammonium hydroxide in ratio 85∶15∶1, respectively. Particular bands on silica plate were captured with Phosphoimager. The activity were calculated by densitometric analysis and normalized by the protein concentration of the tissue homogenate.

### Real-Time PCR

Real time q-rtPCR for CerS were performed as described [3]. Briefly, lungs were harvested from 6- to 8-week-old mice. RNA was isolated using a PerfectPure RNA kit according to manufacturer's instructions, which included a DNase step. cDNA synthesis was performed using a Reverse-iT first strand synthesis kit using random decamers with a 30 min incubation at 42°C and then at 47°C. Total RNA (100 ng) was used to determine expression levels of mouse CerS mRNA, using TaqMan™ analysis and a 7300 Sequence Detection System (Applied Biosystems). Detailed information on primers used is provided in [3]. To control for variability of RNA input, all PCR reactions were normalized to the amount of hypoxanthine guanine phosphoribosyltransferase-1 mRNA.

### Lung Histology

Standardized lung inflation, fixation, and automated morphometric analysis on coded slides were performed as described [14]. Briefly, slides containing 4 µm sections of the paraffin-embedded lung were deparaffinized in xylene, followed by hydration and staining with hematoxylin and eosin.

### Lung X-Gal Staining

The CerS2 promoter activity in CerS2^−/+^ mice was monitored by X-gal staining of lung tissue and sections [15]. Left lobes were inflated with 0.7 ml fixative solution (100 mM phosphate buffer, pH 7.3, 2.5% (v/v) formalin, 0.25% (v/v) glutaraldehyde, 2 mM MgCl_2_, 5 mM EGTA, 0.025% (v/v) NP40). Upon dissection, the left lobes were incubated in the fixative solution for 2 h at 4°C and washed 3 times in 100 mM phosphate buffer, pH 7.3. E. coli β-galactosidase activity was detected by incubation overnight at 37°C in the dark in staining solution (100 mM phosphate buffer, pH 7.3, 5 mM potassium ferricyanide, 5 mM potassium ferrocyanide, 0.01% (v/v) sodium deoxycholate, 0.1% (v/v) NP40, 2 mM MgCl_2_, 1 mg/ml X-gal. After staining, the left lobes were fixed and embedded in paraffin, followed by sectioning and mounting on slides. For imaging, slides were deparaffinized and then a coverslip was mounted on sections, followed by microscopy.

### Statistical Analyses

Statistical analyses was performed with Sigma Stat (Systat Software Inc, Chicago, IL, USA) using an unpaired Student t-test, ANOVA, or Kruskal-Wallis one-way ANOVA on ranks for morphometry analysis. A statistical difference of p<0.05 was considered statistically significant.

## Results

**Figure 2:**
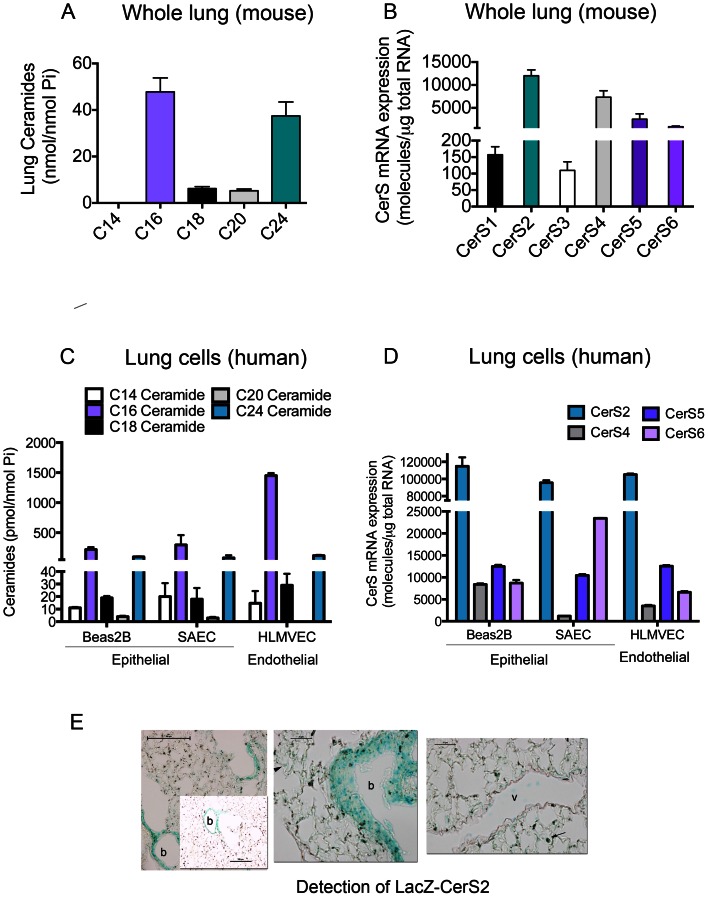
Ceramide levels and CerS expression in the normal lung and human lung cells. A, Levels of ceramide species in the whole mouse lung measured by LC-MS/MS (C57BL/6 mice; female; age 3 months; mean+SEM; n = 5). B, Levels of individual CerS mRNA expressed in the whole mouse lung, measured by real time q-rtPCR; mean+SD, n = 5. C–D, Levels of ceramide species (C) and of CerS mRNA (D) in lung structural cells grown in culture: human bronchial epithelial cell line Beas2B, primary human small airway epithelial cells (SAEC), and primary human microvascular endothelial cells (HLMVEC); means+S.E.M., n = 3. Bar colors of ceramide species corresponding to the color of CerS responsible for its synthesis. E, X-Gal staining (blue) of frozen lung sections from CerS2^−/+^ mice at various magnifications (size bar 100 µm in the left panels and 25 µm in the middle and right panels). Note more prominent transcriptional activity of the LacZ-promoter (blue) in the epithelial layers of the bronchi (b), rather than in the vascular (v) endothelium or alveoli (the arrow indicates an alveolar macrophage).

We first measured the distribution of ceramide species in mouse lung. The most abundant species were C16- and C24- ceramides (both saturated and monounsaturated), which comprised ∼70% of the total ceramides (Fig. 2A). Dihydroceramide, the precursor of ceramide and the initial product of CerS acylation, although 4-fold less abundant than ceramide, showed a similar pattern (data not shown). CerS2 mRNA was the most abundant mRNA in whole lung, followed by CerS4 and CerS5 (Fig. 2B), with CerS6, CerS3 and CerS1 found at much lower levels (Fig. 2B). Since lung tissue homogenates consist of multiple cell types, we analyzed ceramides and CerS in specific human lung cell types. In epithelial cells (a transformed bronchial epithelial cell line, Beas2B, and primary small airway epithelial cells), or in endothelial cells (primary microvascular lung endothelial cells), C16-ceramide was the most abundant, followed by C24:0 and C24:1 (Fig. 2C). Interestingly, compared to epithelial cells, human lung microvascular endothelial cells had markedly increased C16-ceramide levels (Fig. 2C), whereas both lung epithelial and endothelial cells had a similar relative abundance of CerS, with CerS2 mRNA being the highest expressed (Fig. 2D). The localization of CerS2 expression in the whole lung was assessed by staining frozen lung sections for LacZ expression in transgenic CerS2^−/+^ mice. In these mice, CerS2 transcription was measured by a LacZ reporter, such that the higher the CerS2 mRNA levels, the more intense the blue color developed with X-gal staining of the lung. CerS2 transcription was noted primarily in the large airway epithelium, with low levels of expression in the alveolar parenchyma (Fig. 2E).

**Figure 3:**
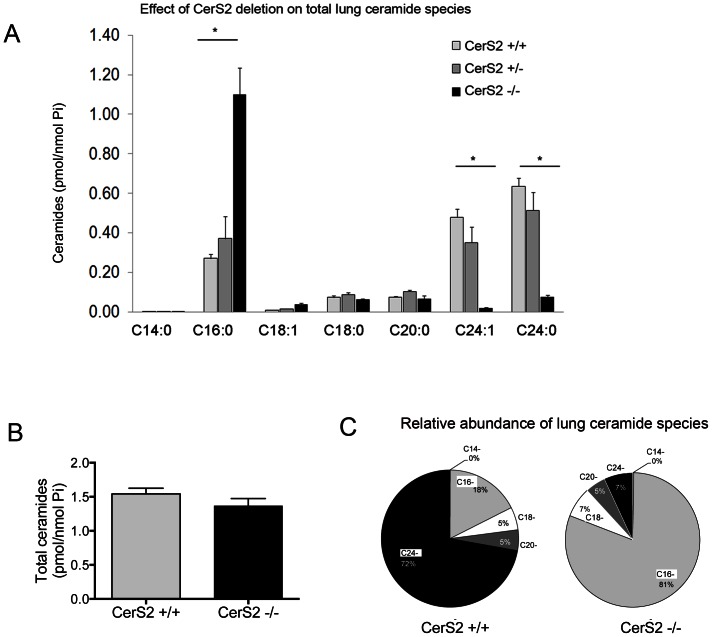
Effect of CerS2 loss of function on lung ceramides. A, Levels of ceramides in the lung of CerS2-null mice (black bars) compared to WT mice (light grey bars) or heterozygous CerS2^−/+^ mice (dark grey bars). Values are means ± S.E.M., n = 3–5; * p<0.05. B, Total ceramide levels in the lungs of CerS2-null mice (black bars) compared to WT mice (light grey bars); values are means ± S.E.M., n = 3–5. C, Relative expression of ceramide species in WT and CerS2-null mice (percent).

We next attempted to delineate the role of CerS2 on ceramide species homeostasis in the lung. As expected, levels of C24:1- and C24:0-ceramides were greatly reduced in CerS2-null mice (Fig. 3A), and similar to other tissues such as liver [10], C16-ceramide levels were markedly elevated (Fig. 3A), such that total ceramide levels were largely unaltered in CerS2-null mice lungs at the time of assessment in these mice (Fig. 3B). Thus, whereas C16-ceramide comprised ∼18% of the total ceramides in the lung of WT mice, it comprised >80% in CerS2-null mice lungs (Fig. 2C). Together, these data suggest that the depletion of CerS2 has multiple effects on ceramide metabolism in lung, similar to those observed in other tissues when CerS2 was inhibited.

**Figure 4:**
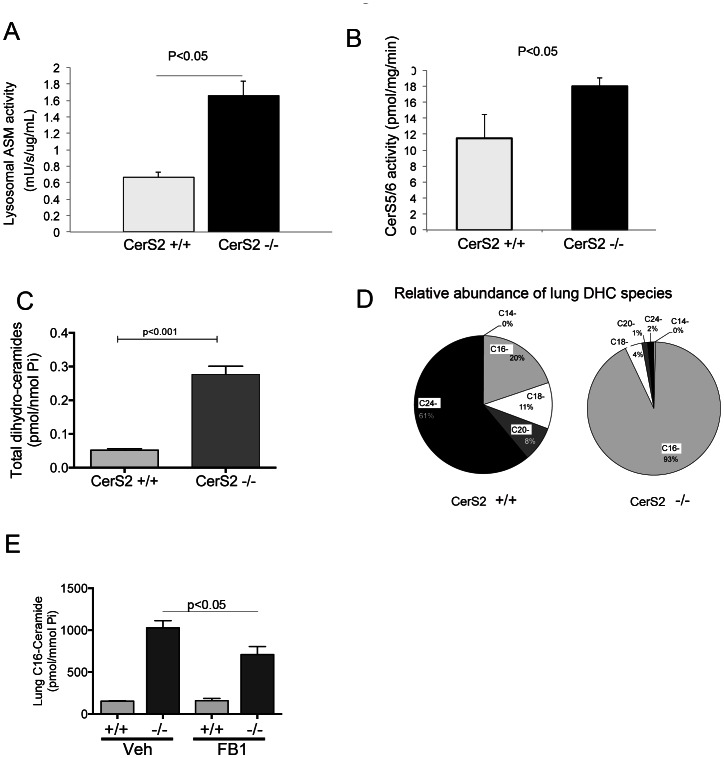
Effect of CerS2 loss of function on sphingolipid metabolic pathways in the lung. A–B, Lung acid sphingomyelinase (lysosomal ASM, A) and CerS 5/6 (B) activities in the whole lung are increased in CerS2-null mice compared to wild type. Mean ± S.E.M., n = 3; * p<0.05. C–D, Total lung dihydroceramide levels (C) are increased in CerS2-null mice, paralleled by marked increases in the abundance of C16-dihydroceramide (D); Mean ± S.E.M., n = 3–7. E, Lung C16-ceramide in mice with indicated CerS2 genotype, following treatment with the general CerS inhibitor FB1 or its vehicle, saline (mean+S.E.M., n = 3).

To understand the impact of CerS2 dysregulation on the lung ceramide homeostasis, we measured ceramide metabolites or enzymes in the sphingolipid pathway in the lungs of CerS2-null mice. The acid sphingomyelinase activity was elevated (Fig. 4A), whereas we could detect no increases in the neutral sphingomyelinase activity (data not shown). Lung ceramide synthase 5/6 activity, responsible for C16-ceramide synthesis was also increased (Fig. 4B), suggesting the de novo and/or recycling pathways were also stimulated in CerS2-null lungs. These changes were associated with higher dihydroceramide levels in the lungs of CerS2-null than in WT mice (Fig. 4C), primarily on account of C16-dihydroceramide (Fig. 4D). These data pointed to an increase of de novo C16-ceramide synthesis, in parallel to that generated by sphingomyelinase hydrolysis. The increases in C16-ceramide and its precursor were noted throughout postnatal lung development (data not shown). To investigate the contribution of CerS5/6 to C16-ceramide levels in CerS2-null mice, we administered a general CerS inhibitor, FB1. Following 3 days of systemic FB1 administration, lung C16-ceramide levels significantly declined by approximately 30%, implicating a compensatory upregulation of the de novo pathway of ceramide synthesis in transgenic mice devoid of VLC ceramides (Fig. 4E).

**Figure 5:**
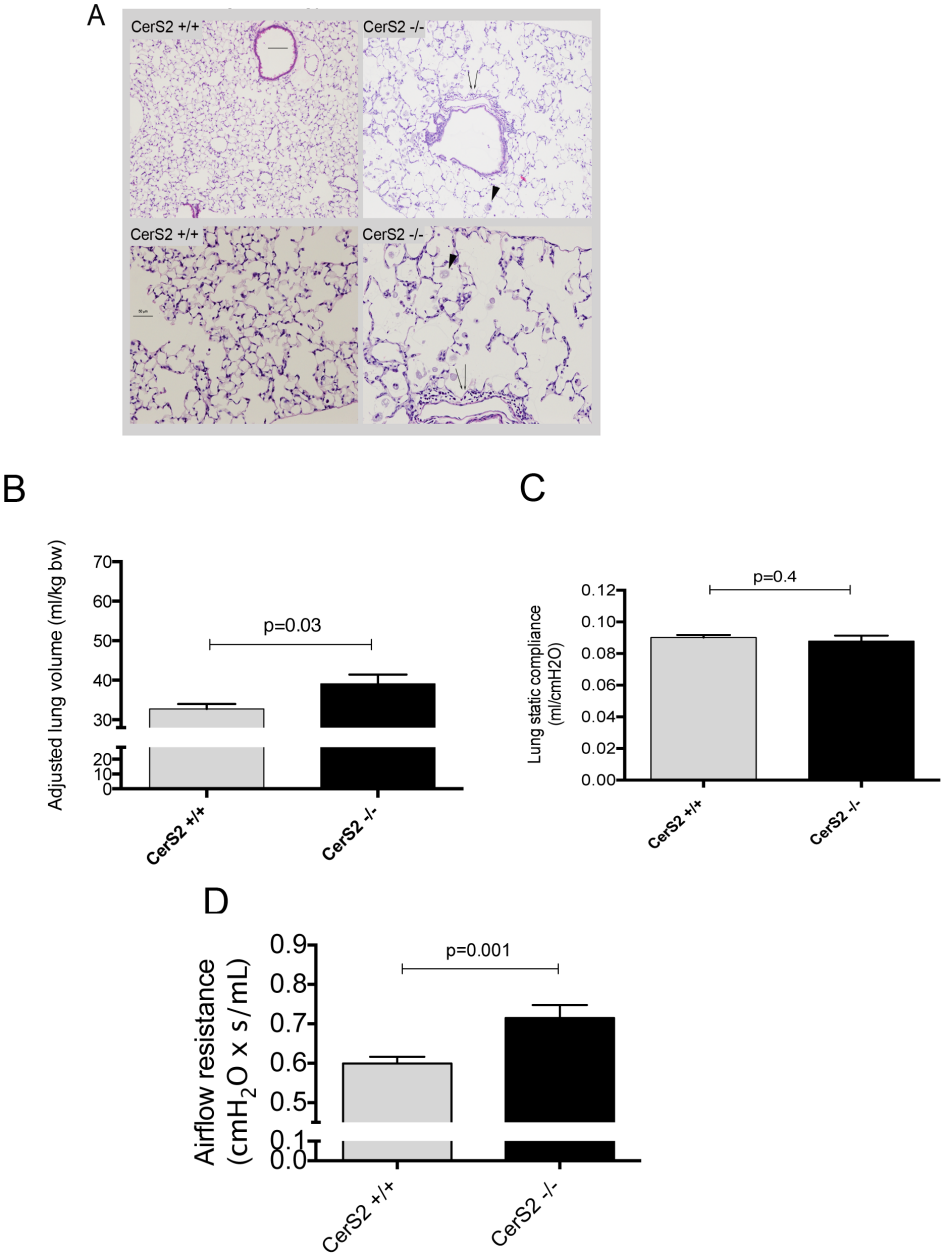
Lung histology and function of CerS2-null mice. A–C, Histological changes in the lung parenchyma and bronchi detected by H & E staining of lung sections from CerS2-null and WT mice (A). Note areas of foamy macrophage infiltration (arrowhead), and areas of inflammation. B–D, Lung function measured by lung volumes adjusted by body weight (B), lung compliance (C), and airflow resistance (D) in WT mice or CerS2-null mice; means+S.E.M., n = 5–14.

To determine the role of CerS2 on lung structure and function, lung histology was examined by hematoxylin-eosin staining of lungs inflated under constant pressure (Fig. 5A–C). Lungs from adult mice (3 months or older) displayed patchy areas of perivascular inflammation, as well as accumulation of foamy alveolar macrophages in the airspaces (Fig. 5A and 5B, inset). To measure the impact of these inflammatory changes on lung function, mice were tested using the Flexivent system. The lack of CerS2 led to mildly increased lung volumes (Fig. 5B), albeit the static lung compliance was not affected (Fig. 5C). However, we noted a significant increase in airflow resistance in CerS2-null mice compared to WT mice (Fig. 5D).

**Figure 6:**
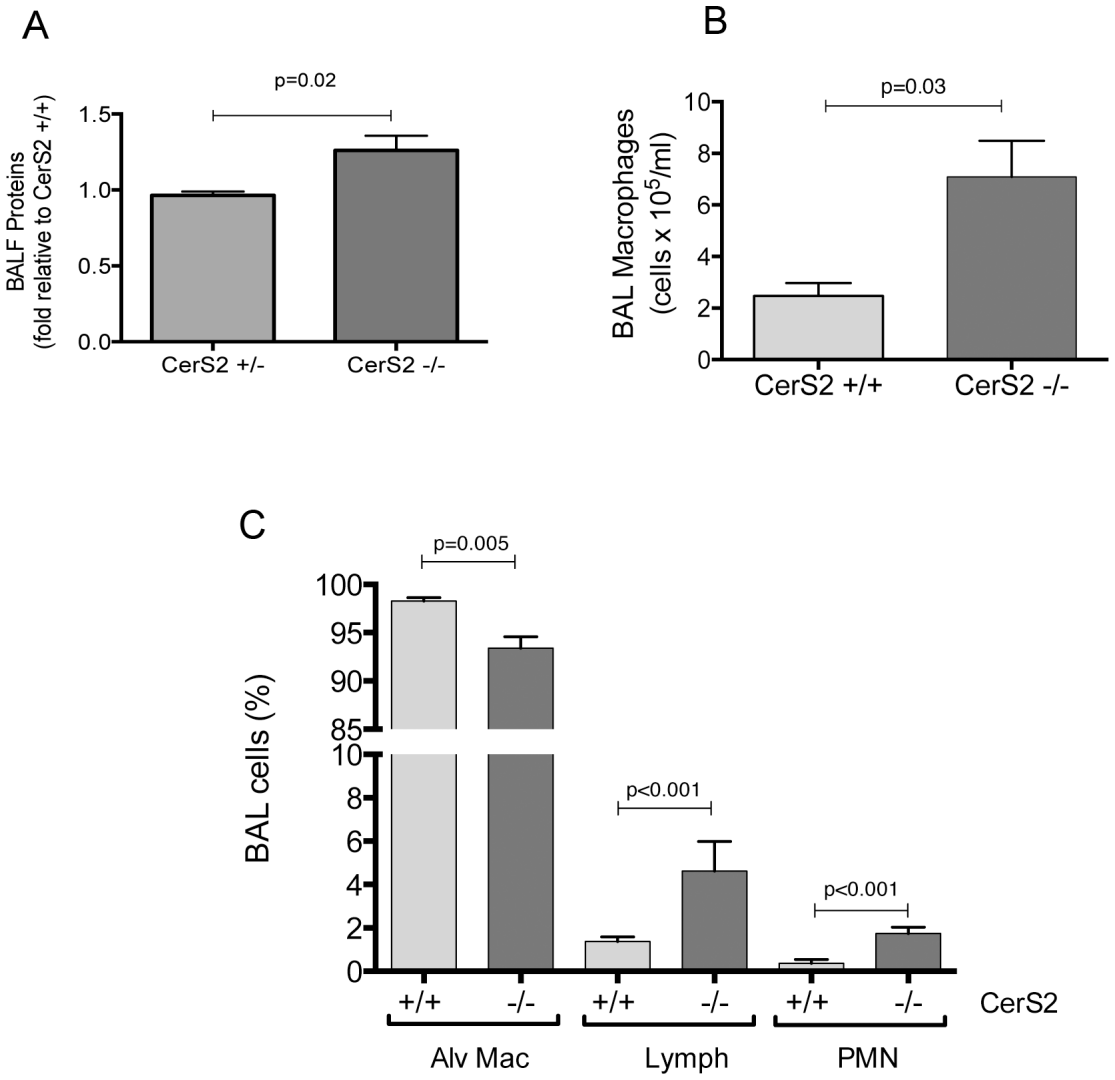
Markers of lung remodeling and inflammation in CerS2-null mice lungs. A–C. Lung inflammation measured by BAL fluid protein content (A, expressed relative to WT control; mean+SEM) and inflammatory cell counts; macrophage numbers (B) and abundance (percent, C) of inflammatory cell macrophages (Mac), lymphocytes (Lym) and polymorphonuclear cells (PMN) in the BAL fluid of WT (light grey) or CerS2-null (black bars) mice, measured by counting on Giemsa-stained cytospin slides; means+S.D., n = 5.

To determine if these functional changes were associated with markers of airway inflammation, we investigated the BAL fluid protein content and cellularity. The BAL fluid from CerS2-null mice exhibited an approximately 25% increase in protein content compared to wild type mice (Fig. 6A) and increased inflammatory cell content. Both the absolute number of alveolar macrophages in the BAL fluid (Fig. 6B), as well as the percentage of lymphocytes and neutrophils were elevated in the airways of CerS2-null mice when compared to wild type mice (Fig. 6C).

## Discussion

We identified that CerS2 and CerS5 are the most abundant CerS in the lung and CerS2 is necessary for proper lung homeostasis. In particular, CerS2 may be essential for the regulation of lung inflammatory cell homeostasis and the maintenance of the functional integrity of airways and lung airspaces. The molecular mechanisms that account for airway inflammation and increased airflow resistance in CerS2-null mice are not known. The abnormally high C16-ceramide, loss of C24-ceramides, elevated dihydroceramide or sphinganine levels (unpublished data), or a combination of sphingolipid metabolite accumulation may all be implicated. These in turn, could directly trigger oxidative stress, apoptosis, may alter inflammatory cell trafficking, or alter host-environment interactions. For example, pathological changes in the liver may be caused by chronic oxidative stress, since levels of several anti-oxidant enzymes are elevated, as is lipid peroxidation, protein nitrosylation, and ROS in the liver of CerS2-null mice [16]. The dysmyelinating phenotype of CerS2 deficiency in the brain may be attributed to the reduction in levels of a glycolipid that is enriched in myelin, a conclusion which was based on the high expression levels of CerS2 in oligodendrocytes [17]. In contrast, our investigations of whole lungs and of lung epithelial and endothelial cells indicated similar relative distribution of CerS isoforms and ceramide species, with the exception of microvascular endothelial cells that had higher absolute levels of C16 ceramide compared to lung epithelial cells. In separate experiments, alveolar macrophages also exhibited higher levels of C24 and C16, compared to other ceramide species (data not shown). These data render it difficult to attribute the lung pathology to the function of ceramides in a specific cell type, but the predominant localization of CerS2 transcription in the adult murine lung and the presence of airflow obstruction suggests epithelial CerS2 may be required for proper airway function.

It is compelling to invoke the effects of the massively upregulated C16 for the airway inflammation and increased airflow resistance in CerS2-null mice. We have recently shown that direct C16-ceramide augmentation, similar to C12-ceramide augmentation in the lungs via single intra-tracheal delivery, increased airway inflammation and oxidative stress, and caused airflow obstruction, which albeit of mild amplitude, was notable even after only several days of C16-ceramide increase [18]. Of note, our mice were not bred and maintained in a pathogen-free facility, and therefore the phenotype of CerS2-null mice may reflect an interaction of the environment (e.g. pathogens) with host factors (e.g. increased C16-ceramide).

Our findings strongly implicate that a balance of VLC- and LC-ceramides is necessary for proper lung homeostasis. The clinical relevance of the observed CerS2-null mouse phenotype may relate to obstructive airways diseases. We noted that CerS2 SNPs may be nominally associated with asthma in a GWAS study [19]. Importantly, this and another GWAS study identified ORMDL, the mammalian form of ORM, which encodes for an enzyme upstream of CerS2 in the de novo pathway of ceramide synthesis [4], to be genome-wide significantly associated with the risk of asthma, a chronic airflow obstructive disease associated with airway inflammation.

In conclusion, we describe the first quantitative data of CerS expression in the lung and association with ceramide species expression in the mouse lung and human lung epithelial and endothelial cells. Our work also addresses for the first time the functional role of any CerS in the lung, implicating CerS2, the enzyme necessary to synthesize very long chain sphingolipids, as an essential molecule for the maintenance of lung airway function. Although it is difficult to pinpoint whether the loss of C24- or the marked increase of C16-ceramides is to blame for the abnormal lung phenotype, C16-ceramide emerges as a likely culprit of lung injury, as corroborated by our recently published work of lung damage caused by selective C16-ceramide delivery to the lung [18]. Another significant contribution of our report is the realization that an abnormal sphingolipid balance is sufficient to increase lung inflammation and trigger tissue remodeling. This illustrates the importance of determining ceramide species in experimental models of lung disease, as previously suggested by observational studies of altered species distribution during ceramide-dependent lung cell apoptosis [12]. Finally, CerS2 deficiency alone was associated with increased airways resistance, which in light of recent genomic studies of asthma could provide useful functional significance of the de novo sphingolipid pathway in this common disease.
